# Memantine, Donepezil, or Combination Therapy—What is the best therapy for Alzheimer’s Disease? A Network Meta‐Analysis

**DOI:** 10.1002/brb3.1831

**Published:** 2020-09-10

**Authors:** Jiaxun Guo, Zhenyu Wang, Ruishu Liu, Yunxia Huang, Nan Zhang, Ruihan Zhang

**Affiliations:** ^1^ Department of Rehabilitation Medicine Yongchuan Hospital Chongqing Medical University Chongqing China; ^2^ Department of Dermatology Yongchuan Hospital Chongqing Medical University Chongqing China

**Keywords:** Acceptability of healthcare, Activities of daily living, Alzheimer disease, Cognitive function, Cost effectiveness, Disease progress

## Abstract

**Introduction:**

Alzheimer's disease (AD) is a degenerative brain disease that progresses over time, heavily burdening patients, families, and aging societies worldwide. Memantine and donepezil are frequently used in its treatment, both as monotherapy and in combination. This multiple treatment comparison meta‐analysis assessed the efficacy of these regimens and placebo in the management of AD.

**Methods:**

We searched PubMed, Embase, the Cochrane Library, and Wanfang Med Online and China National Knowledge Infrastructure for English and Chinese publications from the first records to 17 April 2020. Two investigators scanned articles for placebo‐controlled trials of memantine and donepezil alone and in combination. We extracted data on the following outcomes: cognition, global assessment, daily activities, neuropsychiatric symptoms, adverse events, and the acceptability and cost of these treatment regimens.

**Results:**

Of 936 records screened, we included 54 trials in this analysis. The combination therapy was more effective in improving cognition (mean difference (MD)‐5.01, 95% credible interval (95% Crl) −10.73 to 0.86 in the Alzheimer's Disease Assessment Scale‐Cognitive Subscale; MD 9.61, 95% Crl 2.29 to 16.97 in the Severe Impairment Battery), global assessment (MD −2.88, 95% Crl −6.04 to 0.40), daily activities (MD 13.06, 95% Crl −34.04 to 58.92), and neuropsychiatric symptoms (MD −6.84, 95% Crl −10.62 to –2.82) compared with placebo. Memantine was more acceptable than placebo (MD 0.93, 95% Crl 0.69 to 1.22).

**Conclusions:**

Memantine plus donepezil showed superior outcomes for cognition, global assessment, daily activities, and neuropsychiatric symptoms, but lower acceptability than monotherapy and placebo. Combination therapy may be more cost‐effective, because memantine slows the progression of AD.

## Graphical abstract

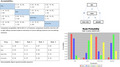

## 1 INTRODUCTION

With aging populations worldwide, the prevalence of chronic diseases, including Alzheimer's disease (AD), increases continuously. Alzheimer's disease (AD), the most common cause of dementia (Alzheimer’s Association, 2020), heavily burdening patients, families, and aging societies worldwide. According to the “World Alzheimer Report, 2015: The Global Impact of Dementia (World Alzheimer Report, 2015),” over 35 million people currently live with AD worldwide. Moreover, the number of patients is estimated to rise to 60 million by 2050 (Ansari, Satar, Perveen, & Ashraf, 2017). AD is a chronic degenerative brain disease, but its cause is not entirely clear at this point. Symptoms of AD include memory loss, difficulty in completing familiar tasks, problems in understanding visual images and spatial relationships, and mood and personality changes, among others(Alzheimer’s Association, 2019). Current studies (Albert et al., 2011; Sperling et al., 2011; Tao et al., 2020) show that before patients are diagnosed with AD, they have experienced a long period of preclinical AD, which is considered to be a critical phase for therapeutic interventions. The pathological cascade does not synchronize with the emergence of clinical symptoms of AD. However, some individuals with the pathological cascade process may not progress to AD. Before AD, there is also the stage of mild cognitive impairment (MCI) of variable length and means that patients will live for many years with this disability before death. Furthermore, there is no must of MCI progressing to AD, and it increases the risk for AD.

Thus, it is difficult to diagnose the condition during the preclinical phase, and many AD patients get diagnosed only during the time of symptomatic predementia phase, which also refer to MCI due to AD. Unfortunately, all of the currently available pharmacologic treatments are easing rather than curing the symptoms. Donepezil, an acetylcholinesterase inhibitor (AChEI), is supported by sufficient data (Haake, Nguyen, Friedman, Chakkamparambil, & Grossberg, 2020) to prove its effect of symptomatic treatment. Memantine, an N‐methyl‐D‐aspartate (NMDA) receptor antagonist, has also been confirmed to be effective in improving memory, awareness, and daily activities of AD patients (Conway, 2020).

The U.S. Food and Drug Administration (FDA) has approved the use of Namzaric® (Allergan Inc., Dublin, Ireland), a combination of donepezil and memantine as an extended release preparation for the combination therapy of patients with moderate‐to‐severe AD. However, the European Medicines Agency (EMA) declined the approval of Acrescent (Lundbeck Inc., Copenhagen, Denmark), a fixed‐dose combination of memantine hydrochloride and donepezil hydrochloride for use in moderate‐to‐severe AD because of a lack of evidence for the effectiveness of the combination therapy (Calhoun, King, Khoury, & Grossberg, 2018; Withdrawal assessment report, 2012).

Some studies (Ashraf et al., 2019; Marta, Katarzyna, & Jerzy, 2017; Sung, Lin, Liu, Su, & Tsai, 2020; Yoshiyama, Kojima, Ishikawa, & Arai, 2010） found indications of a potential infectious agents and chronic inflammation of AD and consecutively sought to treat AD patients with antimicrobial therapy and anti‐inflammatory therapeutics. However, because of the limited range of antiviral preparations and the immaturity of these theories, this approach is not widely used in clinical. Thus, these traditional antidementia medications are still of great significance in the clinical setting.

Therefore, we performed a systematic review and network meta‐analysis to compare the effect, acceptability, adverse events, and cost of memantine and donepezil, both given individually and in combination, with the aim to provide a better choice or new approach for the treatment of AD patients.

## 2 METHODS

### 2.1 Search strategy

We searched for “Alzheimer disease”, “Alzheimer's disease”, “Alzheimer Dementia” and “memantine” and “donepezil” as medical subject headings/keywords in the databases PubMed, Embase, the Cochrane Library, Wanfang Med Online (one of the most used Chinese databases), and China National Knowledge Infrastructure (CNKI, another frequently used Chinese database) from the first record to April 17, 2020. Furthermore, we identified additional articles from the reference lists of the articles to avoid the omission of relevant results.

### 2.2 Selection of studies

**Figure 1:**
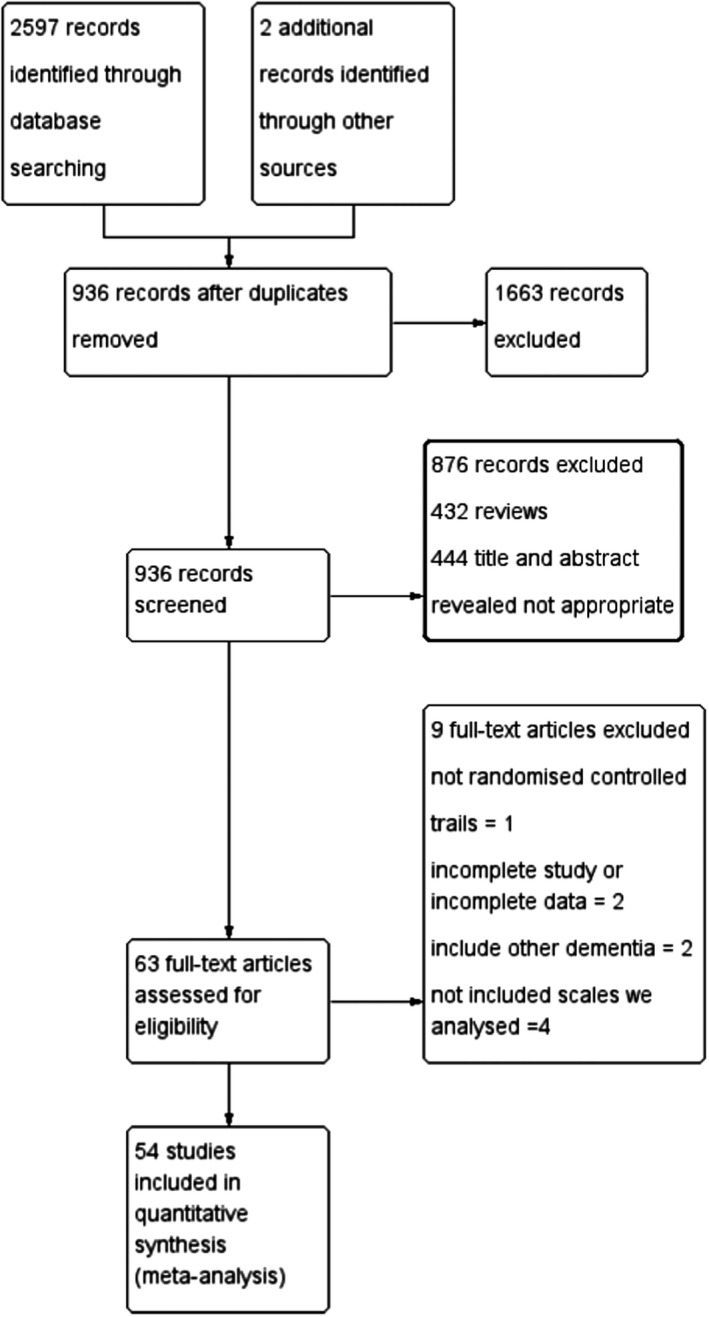
Study selection for a network meta‐analysis on Alzheimer's disease treatment (flow diagram)

The selection process of studies is illustrated in Figure 1

Two investigators independently scanned the titles and abstracts of the identified 936 studies that remained after eliminating duplicates and then read the full text intensively to select the articles meeting the inclusion criteria.

### 2.3 Selection criteria

The inclusion criteria for randomized controlled trials (RCTs) in this systematic review were as follows:

1. published in either English or Chinese;
2. double‐blind or single‐blind trials;
3. endpoints had been published or could be retrieved online;
4. inclusion of patients diagnosed with AD or suspected AD according to the following scales (Fratiglioni, Grut, Forsell, Viitanen, & Winblad, 1992; McKhann et al., 1984; Schneider, Arvanitakis, Leurgans, & Bennett, 2009): the Diagnostic and Statistical Manual of Mental Disorders, National Institute of Neurological and Communicative Disorders and Strokes‐Alzheimer's Disease and Related Disorders Association criteria (NINCDS‐ADRDA).

Exclusion criteria:

1. studies were not RCTs, or RCTs without endpoints;
2. patients with other dementia, such as Parkinson disease‐induced dementia;
3. patients who did not have stable vital signs.

A third investigator would participate when the two primary investigators disagreed about the inclusion of a study. We describe the specific characteristics of the eventually included 54 studies in the Appendix.

### 2.4 Data extraction and quality assessment

Two investigators extracted the basic study characteristics from the full articles independently, and the quality of the included studies was assessed by the computer program Review Manager (RevMan), version 5.3 (Copenhagen: The Nordic Cochrane Centre, The Cochrane Collaboration, 2014). The basic characteristics included the sample size, severity of AD, mean age of patients, medication administered, treatment duration, outcome measures, costs of therapy, and adverse events.

### 2.5 Risk of bias assessment

**Figure 2:**
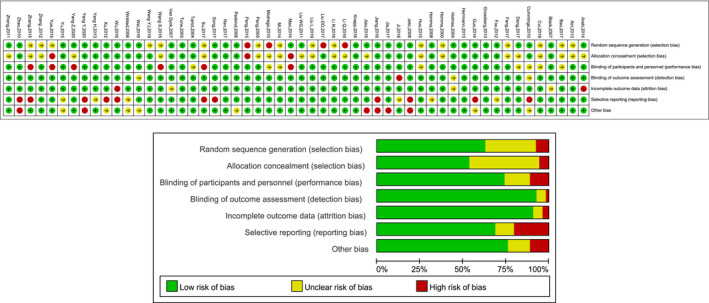
Risk of bias in studies selected for a network meta‐analysis on Alzheimer's disease treatment. Summary based on Review Manager, version 5.3 (Copenhagen: The Nordic Cochrane Centre, The Cochrane Collaboration, 2014) assessment

The risk of bias graph and summary as analyzed by the RevMan 5.3 software are shown in Figure 2

. Most of the 54 included trials utilized a suitable method to minimize bias and were considered to have a low risk of selection bias, performance bias, detection bias, attrition bias, reporting bias, and other bias. Four studies of 54 included studies were rated as having a high risk of bias in the generation of their random allocation scheme. Three trials of 54 included studies did not sufficiently conceal the allocation of treatment to patients. Six trials had a high risk of bias in blinding of participants and personnel, one trial for the blinding of outcome data, and another one for incomplete outcome data. Eleven studies of 54 included studies had a high risk of reporting bias because of incomplete outcomes and six studies for other biases.

### 2.6 Outcomes

The outcomes of this study included the efficacy, acceptability, and costs of therapy. Considering the characteristics of AD, we divided efficacy into four aspects: cognition, global assessment, daily activities, and neuropsychiatric symptoms. Outcomes were assessed using Alzheimer's Disease Assessment Scale‐cognition subscale (ADAS‐cog), the Severe Impairment Battery (SIB) for cognition, the change in the clinical global impression (CGI) for the overall assessment, Alzheimer's Disease Cooperative Study‐Activities of Daily Living (ADCS‐ADL) and Activities of Daily Living (ADL) for daily activities, and the Neuropsychiatric Inventory (NPI) for neuropsychiatric symptoms (Farlow et al., 2013; Panisset, Roudier, Saxton, & Boller, 1994).

Acceptability was measured as treatment discontinuation for any reason cause the dropouts referred to different circumstances such as unsatisfied with treatment, deterioration of patient's condition, or unbearable adverse events.

Costs discussed in the conclusion involved both the direct costs of treatment and caregivers and the indirect costs, such as inability to work.

A validated method was used in the assessment of trials with a lack of information or specific data (Furukawa, Cipriani, Barbui, Brambilla, & Watanabe, 2005; Zhang, Kang, & Chen, 2016).

### 2.7 Data analysis

We conducted a network meta‐analysis to compare the efficacy as the mean and standard deviation (*SD*), assuming that the heterogeneity of each included trial was comparable and ran both a consistency model and inconsistency model. By comparing the potential scale reduction factor(PSRF)of two models, and the median (with 95% credible interval (Crl)) between random effects standard deviation and inconsistency standard deviation to test and verify the choose of consistency model.

Secondly, we chose pooled odds ratios (ORs) to assess acceptability because of the dichotomy of results. The domains were pooled separately, and different type of data would not be pooled for the lack of comparability. The network meta‐analysis was accomplished through ADDIS software (ADDIS Software PLC, Addis Ababa, Ethiopia) that was adopted to analyze acceptability.

Finally, we reviewed the observed adverse events of included studies and reported costs from one study.

## 3 RESULTS

### 3.1 Efficacy

#### 3.1.1 Cognition

**Figure 3:**
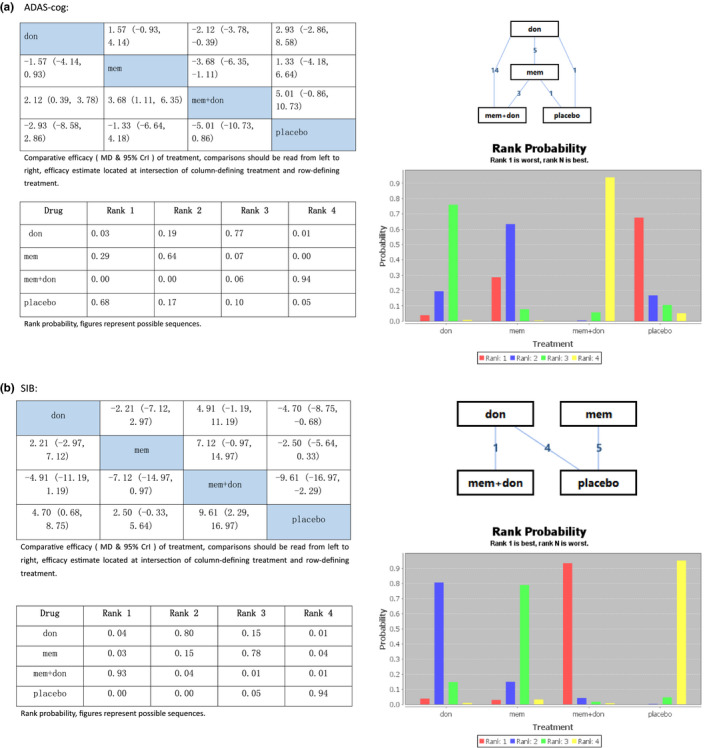
(a) Network meta‐analysis on Alzheimer's disease treatment. Cognition assessment using the Assessment Scale‐cognition subscale. Don, donepezil; mem, memantine; mem + don, combination of donepezil and memantine; ADAS‐cog, Assessment Scale‐cognition subscale; MD, mean difference; Crl, credible interval. (b) Network meta‐analysis on Alzheimer's disease treatment. Cognition assessment using the Severe Impairment Battery. Don, donepezil; mem, memantine; mem + don, combination of donepezil and memantine; SIB, Severe Impairment Battery; MD, standardized mean difference; Crl, credible interval

The PSRFs of both the consistency and inconsistency model for the scale were 1, and the median (95% Crl) of the random effects *SD* and inconsistency *SD* was close to each other, which illustrated the relative effect of each included trial. Therefore, we chose the consistency model for the analysis. The combination treatment of memantine plus donepezil, memantine alone, and donepezil alone all showed a statistically significant difference compared with placebo in both the ADAS‐cog and SIB scales. The ADAS‐cog showed improvement of scores reduced. From the rank possibility that the combination therapy was in the forth rank (MD5.01, 95% Crl −0.86 to 10.73), followed by donepezil in the third rank (MD2.93, 95% Crl −2.86 to 8.58), and memantine was in the second rank (MD1.33, 95% Crl −4.18 to 6.64) than placebo, as illustrated in Figure 3a.

Thus, we concluded that lower rank means the better effect. Therefore, the combination therapy was better than donepezil or memantine monotherapy, and placebo showed the worst effect. In contrast, the SIB scale in Figure 3b showed improvement in the higher scores, so memantine plus donepezil was in the first rank (MD9.61, 95% Crl 2.29 to 16.97), donepezil was in the second rank (MD4.70, 95% Crl 0.68 to 8.75), and memantine was third rank (MD2.50, 95% Crl −0.33 to 5.64) other than placebo, which reflecting the same findings as the ADAS‐cog.

#### 3.1.2 Global assessment

**Figure 4:**
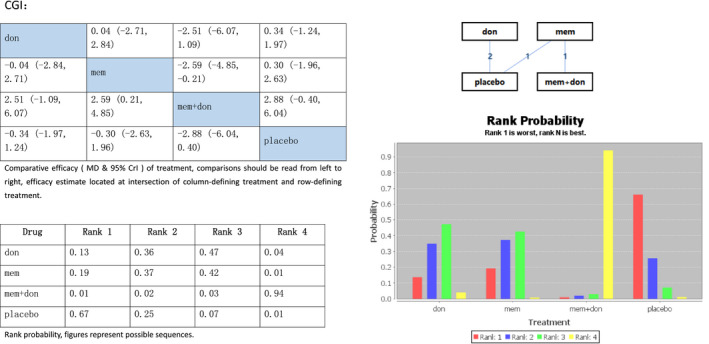
Network meta‐analysis on Alzheimer's disease treatment. Assessment of the change in the clinical global impression. Don, donepezil; mem, memantine; mem + don, combination of donepezil and memantine; CGI, clinical global impression; MD, standardized mean difference; Crl, credible interval

We used the change in the CGI for the global assessment of AD and reducing scores presented treatment effect. Therefore, lower rank means better effect. The consistency model was appropriate for a PSRF of 1, and the median (95% Crl) of the random effects *SD* and inconsistency model *SD* was close to each other. Figure 4 shows that combination therapy in rank 4 was more effective than donepezil (rank 3 (MD‐2.51, 95% Crl −6.07 to 1.09)) or memantine (rank 2.

(MD‐2.59, 95% Crl −4.85 to −0.21)) alone, and placebo was in first rank (MD2.88 95% Crl −0.40 to 6.04), worse than combination therapy.

#### 3.1.3 Daily activities

**Figure 5:**
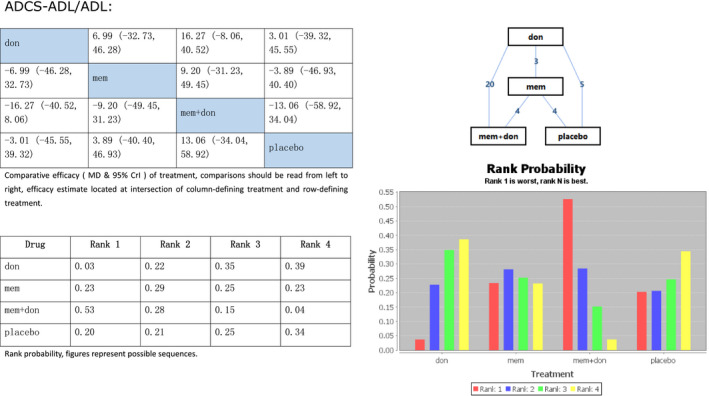
Network meta‐analysis on Alzheimer's disease treatment. Assessment of Alzheimer's Disease Cooperative Study‐Activities of Daily Living and Activities of Daily Living. Don, donepezil; mem, memantine; mem + don, combination of donepezil and memantine; ADCS‐ADL, Alzheimer's Disease Cooperative Study‐Activities of Daily Living; ADL, Activities of Daily Living; MD, standardized mean difference; Crl, credible interval

Some trials chose ADCS‐ADL and others ADL to evaluate the effect of treatment on daily activities in AD. To expand the sample size, we merged two scales into one. We ran a node split and found that the p‐values of all comparisons were above 0.05, and indirect effects were all zero. Therefore, we chose the consistency model that showed in Figure 5

that combination therapy was more effective in rank 1(MD16.27, 95% Crl −8.06 to 40.52) than donepezil alone (rank 4) and memantine more effective in rank 2(MD3.89, 95% Crl −40.40 to 46.93) than placebo.

#### 3.1.4 Neuropsychiatric symptoms

**Figure 6:**
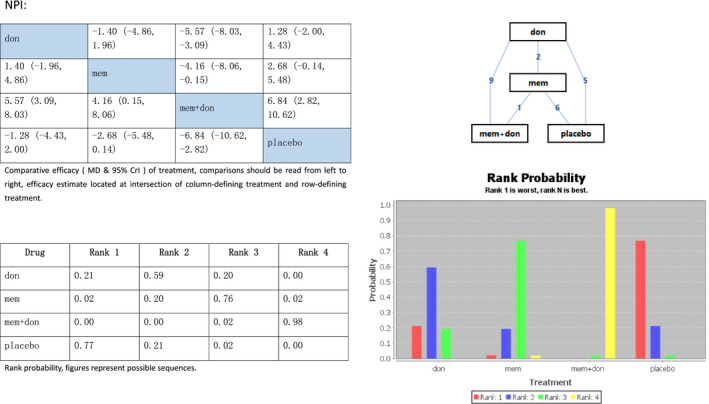
Network meta‐analysis on Alzheimer's disease treatment. Assessment of Neuropsychiatric Inventory. Don, donepezil; mem, memantine; mem + don, combination of donepezil and memantine; NPI, Neuropsychiatric Inventory; MD, standardized mean difference; Crl, credible interval

NPI was accomplished by caregivers that evaluated the neuropsychiatric symptoms of AD patients, as well as the difficulties in caring patients, reducing grades indicated improvement of symptoms and fewer difficulties in caring. The NPI accorded with the consistency model, and the result was exhibited in Figure 6

. Combination therapy was in first rank (MD‐4.16, 95% Crl −8.06 to –0.15) more effective than memantine alone, memantine was in rank 2 (MD −1.40, 95% Crl −4.86 to–1.96) more effective than donepezil, and donepezil was in rank 3 (MD −1.28, 95% Crl −4.43 to –2.00) more effective than placebo in rank 4.

### 3.2 Acceptability

**Figure 7:**
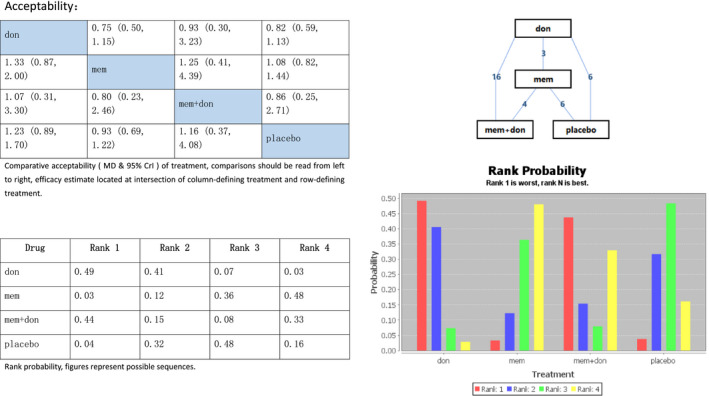
Network meta‐analysis on Alzheimer's disease treatment. Analysis of acceptability. Don, donepezil; mem, memantine; mem + don, combination of donepezil and memantine. MD, standardized mean difference; Crl, credible interval

The PSRF of the consistency model ranged from 1.01 to 1.03, but all were below 1.05, which was tolerable. The differences between the random effects *SD* and inconsistency model *SD* were acceptable, and we adopted the consistency model. Figure 7

shows that memantine had higher acceptability (MD 0.93, 95% Crl 0.69 to 1.22) than placebo, while the difference between donepezil and combination therapy was small (MD 1.07, 95% Crl 0.31 to 3.3).

### 3.3 Costs

One study (Martin et al., 2017) had performed a cost‐effectiveness analysis comparing donepezil, memantine, donepezil plus memantine, and placebo. Martin et al. compared outcomes and costs of 295 community‐dwelling patients with moderate‐to‐severe AD after 52 weeks and found if one ignored the cost‐effectiveness model and only focused on the unadjusted costs (consisting of health and social care costs), costs were highest in the placebo group at a total of £7,964 per person per year, followed by memantine plus donepezil at £5,892, and donepezil alone at £5,418. Memantine costs were lowest at £4,864. When taking into account effectiveness, the combination therapy showed no superiority in cognition, daily activities, and quality of life than memantine or donepezil alone calculated by The National Institute for Health and Care Excellence (NICE) thresholds for Quality‐adjusted life years (QALY) gains.

### 3.4 Adverse events

Adverse events were similar across the 54 included trials. They manifested as dizziness, agitation, confusional state, diarrhea, falls, and emotional problems, among others. No adverse events were related to death.

## 4 DISCUSSION

This network meta‐analysis of 54 studies, conducted in Asia, North America, and Europe, included patients randomly assigned to donepezil, memantine, donepezil–memantine, and placebo. It considered both English and Chinese publications, which expanded the scope and made the conclusions more reliable than an analysis of English articles only. The combination therapy of donepezil and memantine was most effective in improving cognition, global assessment, activities of daily living, and neuropsychiatric symptoms in AD patients (mostly moderate‐to‐severe AD), and the acceptability was slightly higher than that of donepezil and lower than that of memantine.

A previous review (Calhoun et al., 2018) found that the combination of donepezil–memantine had benefits in clinical efficacy over donepezil alone, although it was more expensive than monotherapy. Other two reviews (Farrimond, Roberts, & McShane, 2012; Gauthier & Molinuevo, 2013) reviewed the efficacy in cognition, daily activities, global assessment, and burden of care of adding memantine to different AChEIs (donepezil was one of these AChEIs) and drew positive conclusions. A meta‐analysis by Chen et al. (Chen et al., 2017) found that memantine combined with donepezil had better effects on cognitive and overall function and behavioral and psychological symptoms than donepezil alone. Our network meta‐analysis involved studies until 2020 in both English and Chinese and also took memantine into analysis.

The ADAS‐cog and SIB are both used to measure the severity of AD.

The ADAS‐cog is usually used in mild or moderate and even preclinical AD, whereas the SIB is more frequently used in severe dementia (Kueper, Speechley, & Montero‐Odasso, 2018; Qazi et al., 2005). We adopted both the ADAS‐cog and SIB scales in this meta‐analysis, and the combination therapy of memantine plus donepezil showed excellent results for both scales, indicating its efficacy in mild and severe dementia. Donepezil showed an advantage over memantine in mild‐to‐moderate AD, whereas memantine was more effective than donepezil in severe AD.

The combination therapy of memantine plus donepezil achieved better outcomes than placebo in the CGI. The CGI scale combines the severity of illness, global improvement, and efficacy index. Here, the combination of memantine plus donepezil demonstrated its overall superiority to the full extent in clinical global impression.

The ADCS‐ADL and ADL scales contain eating, going to the toilet, bathing, showering, and other skills of daily living. They reflect not only the quality of life of patients but also the quality of life of their caregivers (if existent). The combination of memantine plus donepezil was the most effective regimen in this regard, too, followed by memantine. Donepezil alone was less effective than placebo.

The NPI is one of the most commonly used scales to assess the neuropsychiatric symptoms of AD and is also associated with the progress of dementia (Cummings et al., 1994; Mallo et al., 2020). Neuropsychiatric symptoms present a particularly heavy burden for both patients themselves and their caregivers (Lyketsos et al., 2011). Therefore, the efficacy of the combination of memantine plus donepezil in this aspect may delay the progression in patients with AD. Previous studies (Dou et al., 2018; Trinh, Hoblyn, Mohanty, & Yaffe, 2003) have concluded on the efficacy of the combination of memantine plus AChEIs in improving neuropsychiatric symptoms, and our network meta‐analysis confirmed that memantine plus donepezil is superior to placebo in this regard.

When taking all four AD dimensions investigated in this meta‐analysis into account, we conclude on the superiority of the combination therapy of memantine plus donepezil in the treatment of AD over monotherapy with either of the substances.

All four treatment regimens were well tolerated by most patients. Memantine was the best‐tolerated medication, followed by placebo and the combination of memantine plus donepezil. Donepezil was the least tolerable. That means, in patients with substantial intolerance to the combination of memantine plus donepezil, a change to memantine is a better choice than donepezil.

One study concluded (Knapp et al., 2017) that there is no more cost‐effective treatment than donepezil. We argue that when taking the effectiveness and the slowing of the clinical progression of AD in patients into account (Wilkinson & Andersen., 2007; Wilkinson, Wirth, & Goebel., 2014), the combination therapy is more cost‐effective, even when the costs are slightly higher than those of donepezil alone. Another study (Cappel, Herrmann, Cornish, & Lanctôt, 2010) also found a more positive effect on the cost‐effectiveness of the combination therapy than of donepezil alone.

This network meta‐analysis has some limitations. The quality of some of the included trials was not high, which may have influenced the entire network meta‐analysis. More high‐quality studies are needed to analyze the optimal therapy of AD in the future. Secondly, this study only searched published trials but not the Cochrane Central Register of Controlled Trials or some other databases for the registration of clinical trials, which might result in a publication bias of the meta‐analysis and have overestimated the study outcomes. Furthermore, this study applied ranking for the outcomes, which has a substantial degree of imprecision for most interventions that have a large width of 95% Crls (Trinquart, Attiche, Bafeta, Porcher, & Ravaud, 2016). Finally, we only reviewed the cost‐effectiveness of therapeutic regimens in this meta‐analysis, but future studies should take the slowing of clinical progression into account.

## 5 CONCLUSION

Our study found that the combination of memantine plus donepezil had superior effects on cognitive and neuropsychiatric symptoms, the global assessment, and daily activities, but was less acceptable to patients compared to either memantine alone or placebo. While memantine was most acceptable, the choice of medication should depend on the individual situations of patients. The extent of damage in the four different domains of AD will lead to different treatment decisions. Furthermore, considering the natural course of AD, memantine plus donepezil may be more cost‐effective than donepezil because memantine may slow the progression of AD.

## 6 ETHICS APPROVAL

Not applicable.

## CONFLICT OF INTERESTS

The authors declare that there have no conflicts of interest.

## AUTHORS' CONTRIBUTIONS

JG designed the study, extracted the data with NZ, did meta‐analysis and network meta‐analysis with RZ, and wrote and approved the manuscript. ZW designed the study, revised, and approved the manuscript with RL. YH discussed the validity of the trials and revised the manuscript.

### Peer Review

The peer review history for this article is available at https://publons.com/publon/10.1002/brb3.1831.

## Appendix 1

**Table:**

Study, year	country	Age(years) Mean + *SD*	severity	treatment	efficacy measure scores	Adverse events	dropout
Araki, 2014	Japan	EG: 77.9 ± 9.8 CG: 79.8 ± 4.6	moderate‐to‐severe	EG: memantine+ donepezil CG: memantine	MMSE, CDT, NPI, J‐ZBI, CGI‐I, and NIRS	EG: 3 CG: 0	EG: 7 CG: 5
Atri, 2013	USA	Mod‐to‐ sev: EG: 75.1 ± 8.5, CG: 75.8 ± 8.5 Mod: EG: 75.9 ± 8.4, CG: 76.4 ± 8.2	Moderate‐to‐severe, and moderate	EG: memantine + donepezil CG: placebo + donepezil	SIB/ADCS‐Cog, ADCS‐ADL, CIBIC‐Plus	Mod‐to‐ sev: EG: 206, CG: 186 Mod: EG: 144, CG: 136	_
Black, 2007	Canada	EG: 78.0 ± 8.04 CG: 78.0 ± 8.20	severe	EG: donepezil CG: placebo	SIB, CIBIC‐Plus, MMSE, ADCS‐ADL, NPI	EG: 140 CG: 117	EG: 59 CG: 40
Cummings, 2010	USA	EG: 77.8 ± 8.6 CG: 79.0 ± 8.2	severe	EG: donepezil CG: placebo	MMSE, SIB	_	_
Fox, 2012	UK	EG: 84.9 ± 6.7 CG: 84.4 ± 6.6	Moderate‐to‐severe	EG: memantine CG: placebo	CAMI, NPI, SIB, SMMSE, CGI‐C	EG: 135 CG: 128	EG: 25 CG: 21
Grossberg, 2013	USA	EG: 76.2 ± 8.4 CG: 76.8 ± 7.8	Moderate‐to‐severe	EG: memantine CG: placebo	SIB, CIBIC‐Plus, ADCS‐ADL, NPI, VFT	EG: 214 CG: 214	EG: 69 CG: 63
Herrmann, 2013	Canada	EG: 74.7 ± 7.9 CG: 75.1 ± 6.9	Moderate‐to‐severe	EG: memantine CG: placebo	NPI, SIB	EG: 138 CG: 136	EG: 31 CG: 32
Holmes, 2004	UK	EG: 78.6 ± 1.4 CG: 78.8 ± 1.2	Mild‐to‐moderate	EG: donepezil CG: placebo	NPI, MMSE	_	EG: 6 CG: 10
Homma, 2000	Japan	EG: 70.1 ± 7.6 CG: 69.4 ± 8.8	Mild‐to‐moderate‐severe	EG: donepezil CG: placebo	ADAS‐j, CDR‐SB, MENFIS, CMCS	EG: 54 CG: 33	_
Homma, 2008	Japan	EG 1:78.0 ± 8.9 EG 2:76.9 ± 7.9 CG: 79.7 ± 7.5	Severe	EG 1: donepezil (5 mg/d) EG 2: Donepezil (10 mg/d) CG: placebo	SIB, ADCS‐ADL, CIBIC‐Plus	EG 1:101 EG 2: 96 CG: 105	EG 1: 20 EG 2: 25 CG: 26
Jelic, 2008	Sweden	EG: 84.5 ± 6.0 CG: 85.3 ± 5.9	Severe	EG: donepezil CG: placebo	SIB, ADCS‐ADL, MMSE, NPI, CGI‐I	_	_
Jia, 2017	China	EG: 71.6 ± 8.56 CG: 70.0 ± 9.57	severe	EG: donepezil CG: placebo	SIB, CIBIC‐Plus, MMSE	EG: 53 CG: 45	EG: 29 CG: 30
Knapp, 2016	UK	EG 1:77.2 EG 2:76.2 EG 3:77.5 CG: 77.7	Moderate‐to‐severe	EG 1: Donepezil EG 2: memantine EG 3: donepezil + memantine CG: placebo	sMMSE, BADLS, NPI	_	_
Modrego, 2010	Spain	77.28 ± 4.88	Mild‐to‐moderate	EG 1: donepezil EG 2: memantine	ADCS‐cog, DAD, NPI	_	_
Peng, 2005	China	EG: 72.6 ± 6.8 CG: 71.8 ± 8.2	Mild‐to‐moderate	EG: donepezil CG: placebo	MMSE, CDR, ADL	EG: 7 CG: 2	EG: 4 CG: 2
Peskind, 2006	US	EG: 78.0 ± 7.3 CG: 77.0 ± 8.2	Mild‐to‐moderate	EG: memantine CG: placebo	ADAS‐cog, ADCS‐ADL, CIBIC‐Plus, NPI,	EG: 120 CG: 99	EG: 36 CG: 35
Tariot, 2004	US	EG: 75.5 ± 8.45 CG: 75.5 ± 8.73	Moderate‐to‐severe	EG: memantine CG: placebo	SIB, ADCS‐ADL, CIBIC‐Plus, NPI	EG: 158 CG: 156	EG: 30 CG: 51
Tune, 2003	US	EG: 73.7 CG: 72.2	Mild‐to‐moderate	EG: donepezil CG: placebo	ACAS‐cog, NPI	_	_
Van Dyck, 2007	US	EG: 78.1 ± 8.2 CG: 78.3 ± 7.6	Moderate‐to‐severe	EG: memantine CG: placebo	SIB, ADCS‐ADL, CIBIC‐Plus, NPI	EG: 131 CG: 125	EG: 44 CG: 46
Winblad, 2006	Sweden	EG: 84.5 ± 6.0 CG: 85.3 ± 5.9	Severe	EG: donepezil CG: placebo	SIB, ADCS‐ADL, CGI‐I, MMSE, NPI	EG: 126 CG: 100	EG: 33 CG: 21
Zhang, 2015	China	EG: 69.75 ± 8.06 CG: 70.13 ± 7.99	Mild‐to‐moderate	EG: memantine CG: donepezil	ADAS‐cog, ADL, NPI, MMSE	_	_
Bao, 2017	China	EG: 65.0 ± 7.2 CG: 65.4 ± 6.4	_	EG: donepezil + memantine CG: donepezil	ADL, MMSE, ADAS‐cog,	EG: 7 CG: 8	EG: 0 CG: 0
Cui, 2019	China	EG: 69.7 ± 4.3 CG: 69.9 ± 4.5	_	EG: donepezil + memantine CG: donepezil	ADAS‐cog, ADL, NPI, MMSE	EG: 7 CG: 8	EG: 0 CG: 0
Deng, 2017	China	EG: 66.4 ± 5.4 CG: 67.2 ± 4.9	_	EG: donepezil + memantine CG: memantine	ADAS‐cog, ADL	EG: 2 CG: 3	EG: 0 CG: 0
Fang, 2017	China	EG: 71.25 ± 4.22 CG: 71.23 ± 4.25	‐	EG: donepezil + memantine CG: donepezil	MMAE, ADL	EG: 5 CG: 4	EG: 0 CG: 0
Guo, 2014	China	EG: 74.9 ± 1.02 CG: 75.10 ± 0.98	Moderate‐to‐severe	EG: donepezil + memantine CG: donepezil	SIB, ADL	EG: 6 CG: 5	‐
Huo, 2015	China	EG: 70.3 ± 3.1 CG: 69.3 ± 2.8	Moderate‐to‐severe	EG: donepezil + memantine CG: memantine	MMSE, ADAS‐cog, ADL, NPI	EG: 18 CG: 15	EG: 0 CG: 0
Ji, 2018	China	EG: 68.3 ± 5.4 CG: 70.5 ± 6.2	moderate	EG: memantine CG: donepezil	MMSE, ADL	EG: 1 CG: 0	EG: 0 CG: 0
Jiang, 2016	China	EG: 75.62 ± 8.13 CG: 76.37 ± 5.30	_	EG: donepezil + memantine CG: donepezil	MoCA, MMSE, ADL	_	_
Jiao, 2016	China	EG: 65 ± 6 CG: 67.6 ± 6	_	EG: donepezil + memantine CG: donepezil	ADAS‐cog, MMSE, ADL	EG: 6 CG: 7	EG: 0 CG: 0
Li Q, 2016	China	EG: 85.01 ± 3.31 CG: 82.67 ± 5.44	_	EG: donepezil + memantine CG: donepezil	ADAS‐cog, MMSE, ADL, NPI	EG: 0 CG: 7	_
Li R, 2016	China	EG: 77.5 ± 6.7 CG: 76.2 ± 6.4	Moderate‐to‐severe	EG: donepezil + memantine CG: donepezil	ADAS‐cog, MMSE, ADL, NPI	EG: 3 CG: 0	EG: 0 CG: 0
Liu DD, 2017	China	EG: 76.5 ± 8.2 CG: 77.3 ± 8.1	_	EG: donepezil + memantine CG: donepezil	MMSE, ADL	EG: 2 CG: 1	EG: 0 CG: 0
Liu L, 2019	China	EG: 64.5 ± 3.5 CG: 65.6 ± 4.5	_	EG: donepezil + memantine CG: donepezil	MMSE, ADL	EG: 4 CG: 3	EG: 0 CG: 0
Liu WG, 2011	China	EG 1:77.2 ± 4.7 EG 2:76.4 ± 4.9	_	EG 1: memantine EG 2: donepezil	MMSE	EG 1:8 EG 2:19	EG 1:0 EG 2:0
Mao, 2014	China	68.5 ± 10.0	_	EG: donepezil + memantine CG: memantine	MMSE, ADL	EG: 9 CG: 8	EG: 0 CG: 0
Mi, 2014	China	EG: 74.0 ± 6.7 CG: 74.3 ± 6.7	Moderate‐to‐severe	EG: donepezil + memantine CG: donepezil	ADAS‐cog, MMSE, ADL, NPI	EG: 13 CG: 11	EG: 0 CG: 0
Peng, 2015	China	EG: 83.4 ± 10.5 CG: 82.6 ± 9.6	Moderate‐to‐severe	EG: donepezil + memantine CG: donepezil	ADAS‐cog, MMSE, ADL, NPI	_	EG: 3 CG: 2
Ren, 2017	China	EG 1:72.34 ± 4.76 EG 2:71.94 ± 5.03	Mild‐to‐moderate	EG 1: memantine EG 2: donepezil	MMSE, GDS, ADAS‐cog, ADL	EG 1:7 EG 2:4	EG 1:0 EG 2:0
Song, 2017	China	EG: 70.1 ± 7.0 CG: 70.7 ± 6.6	_	EG: donepezil + memantine CG: donepezil	ADAS‐cog, MMSE, ADL, NPI	EG: 1 CG: 1	_
Su, 2017	China	EG: 73.74 ± 2.06 CG: 7.25 ± 1.24	_	EG: donepezil + memantine CG: donepezil	ADAS‐cog, MMSE, ADL	EG: 6 CG: 7	EG: 0 CG: 0
Wang X, 2015	China	EG: 75.5 ± 6.7 CG: 76.1 ± 6.9	_	EG: donepezil + memantine CG: donepezil	ADCS‐cog, ADL, MMSE	EG: 7 CG: 6	EG: 0 CG: 0
Wang YJ, 2019	China	EG: 73.62 ± 6.43 CG: 73.62 ± 6.43	_	EG: donepezil + memantine CG: donepezil	MMSE	_	EG: 0 CG: 0
Wei, 2016	China	EG: 69.2 ± 8.3 CG: 69.1 ± 8.1	_	EG: donepezil + memantine CG: donepezil	ADAS‐cog, MMSE, ADL, NPI	EG: 2 CG: 3	EG: 0 CG: 0
Wu, 2016	China	EG: 72.31 ± 6.62 CG: 72.51 ± 6.07	_	EG: donepezil + memantine CG: donepezil	ADAS‐cog, ADL	_	_
Xu, 2012	China	_	Moderate‐to‐severe	EG: donepezil + memantine CG: donepezil	MMSE, ADL	_	_
Yang H, 2013	China	EG: 74.9 ± 1.02 CG: 75.1 ± 0.98	Moderate‐to‐severe	EG: donepezil + memantine CG: donepezil	ADL	EG: 6 CG: 5	_
Yang Y, 2020	China	EG: 69.04 ± 1.65 CG: 68.92 ± 1.74	_	EG: donepezil + memantine CG: donepezil	MMSE, ADAS‐cog, ADL	_	_
Yang Z, 2020	China	EG: 80.98 ± 6.81 CG: 80.34 ± 6.58	_	EG: donepezil + memantine CG: donepezil	MMSE, ADL	EG: 7 CG: 16	EG: 0 CG: 0
Yu, 2015	China	EG: 78.7 ± 10.4 CG: 70.4 ± 9.3	_	EG: donepezil + memantine CG: donepezil	MMSE, ADL, NPI	EG: 4 CG: 2	EG: 0 CG: 0
Yue, 2015	China	EG: 77.5 ± 5.8 CG: 72.3 ± 6.4	_	EG: donepezil + memantine CG: donepezil	ADAS‐cog, NPI, ADL	EG: 0 CG: 2	EG: 0 CG: 0
Zhang, 2012	China	EG1: 74.8 ± 8.2 EG2: 75.1 ± 7.9	_	EG1: memantine EG2: donepezil	MMSE, ADAS‐cog	EG1: 1 EG2: 8	EG1: 0 EG2: 0
Zhao, 2010	China	EG1: 70.3 ± 4.9 EG2: 69.1 ± 5.6	_	EG1: memantine EG2: donepezil	MMSE, ADAS‐cog	_	_
Zheng, 2011	China	85.96 ± 4.82	_	EG: donepezil + memantine CG: donepezil	ADAS‐cog, MMSE, ADL, NPI	EG: 2 CG: 2	EG: 0 CG: 0
